# Postoperative Concurrent Chemoradiotherapy *Versus* Radiotherapy Alone for Advanced Oral Cavity Cancer in the Era of Modern Radiation Techniques

**DOI:** 10.3389/fonc.2021.619372

**Published:** 2021-03-12

**Authors:** Tae Hyung Kim, In-Ho Cha, Eun Chang Choi, Hye Ryun Kim, Hyung Jun Kim, Se-Heon Kim, Ki Chang Keum, Chang Geol Lee

**Affiliations:** ^1^ Department of Radiation Oncology, Yonsei Cancer Center, Yonsei University College of Medicine, Seoul, South Korea; ^2^ Department of Radiation Oncology, Eulji General Hospital, College of Medicine, Eulji University, Seoul, South Korea; ^3^ Department of Oral and Maxillofacial Surgery, Yonsei University College of Dentistry, Seoul, South Korea; ^4^ Department of Otorhinolaryngology, Yonsei University College of Medicine, Seoul, South Korea; ^5^ Division of Medical Oncology, Department of Internal Medicine, Yonsei Cancer Center, Yonsei University College of Medicine, Seoul, South Korea

**Keywords:** oral cancer, intensity modulated radiotherapy, chemotherapy, treatment outcome, prognosis

## Abstract

**Background/Purpose:**

Surgery followed by postoperative radiotherapy (RT) has been considered the standard treatment for oral cavity squamous cell carcinoma (OCSCC) of advanced stages or with adverse prognostic factors. In this study, we compared the outcomes in patients with OCSCC who received postoperative concurrent chemoradiotherapy (CCRT) or postoperative RT alone using modern RT techniques.

**Methods:**

A total of 275 patients with OCSCC treated between 2002 and 2018 were retrospectively analyzed. Adverse prognostic factor was defined as extranodal extension (ENE), microscopically involved surgical margin, involvement of ≥2 lymph nodes, perineural disease, and/or lymphovascular invasion (LVI). In total, 148 patients (54%) received CCRT and 127 patients (46%) received RT alone. More patients in the CCRT group had N3 disease and stage IVB disease (46.6% *vs.* 10.2%, *p*<0.001), ENE (56.1% *vs.* 15.7%, *p*<0.001), LVI (28.4% *vs.* 13.4%, *p*=0.033).

**Results:**

With a median follow-up of 40 (range, 5–203) months, there were no significant differences in the 5-year overall survival (OS) and PFS between treatment groups. In the subgroup analysis according to high risk, the concurrent use of chemotherapy showed significantly improved OS in patients with ENE (HR 0.39, *p*=0.003).

**Conclusion:**

Our retrospective study showed that postoperative CCRT group had comparable survival outcomes to those in the RT alone group for advanced OCSCC in the era of modern RT techniques and indicated that concurrent chemotherapy should be administered to patients with ENE. Prospective randomized studies for confirmation are needed.

## Introduction

Squamous cell carcinoma (SCC) is the most common malignancy of the oral cavity  (1). It is estimated that 35,130 people will be diagnosed with oral cavity cancer in 2019  (2). Surgery followed by postoperative radiotherapy (PORT) is considered the standard treatment for oral cavity SCC (OCSCC) of advanced stages or with adverse prognostic factors. In general, patients with OCSCC tend to have worse local and regional control compared to other head and neck subsites  (3–5).

Evidence for the concurrent use of chemotherapy was established by the European Organization for Research and Treatment of Cancer (EORTC) 22931 and Radiation Therapy Oncology Group (RTOG) 9501 trials  (6, 7). Concurrent chemotherapy with cisplatin was administrated for high risk patients and reported that 5-year overall survival (OS) was approximately 50%.

With advances in radiotherapy (RT) techniques, intensity-modulated RT (IMRT) has been the standard RT for head and neck tumors. In this study, we compared the outcomes in patients with oral cavity SCC (OCSCC) who received postoperative concurrent chemoradiotherapy (CCRT) or PORT alone using modern RT techniques.

## Materials and Methods

### Study Population

A list of consecutive patients who were diagnosed with OCSCC and received RT between 2002 and 2018 was extracted from an institutional cancer registry; a total of 486 patients were identified. The inclusion criteria were as follows: pathologically confirmed SCC of oral cavity, resection of primary tumor with/without neck node dissection, and received postoperative RT using three-dimensional conformal RT (3D-CRT) or IMRT. Exclusion criteria were as follows: PORT followed by salvage resection after recurrence of disease (n = 145); pathologically not a SCC, such as adenoid cystic carcinoma or sarcoma (n = 41); palliative treatment due to distant metastasis (n = 21); and OCSCC with double primary lung cancer (n = 4). After all exclusions, the data of 275 patients were analyzed.

The procedures followed in this study were in accordance with the Helsinki Declaration of 1975, as revised in 2000. This study was approved by our Institutional Review Board (IRB # 4-2019-0401).

### Treatment

Pretreatment evaluation included a complete history, physical examination and laboratory studies including a complete blood cell count and serum chemistry profile. Patients underwent imaging studies such as computed tomography (CT), magnetic resonance imaging for primary tumor and neck node involvement evaluation, and positron emission tomography (PET) for systemic evaluation.

The surgical techniques included resection either by open approaches or by trans-oral robotic surgery using da Vinci Robot (Intuitive Surgical Inc., Sunnyvale, CA). Neck dissection was performed on the involved side or both sides of neck in order to examine the regional lymph node involvement.

RT was delivered using megavoltage photons (≥6 MV). Using 3D-CRT, a cone-down technique was used. Using IMRT, the simultaneous integrated boost technique was used in all patients. The high-risk clinical target volume (CTV)1 encompassed the primary tumor bed (based on preoperative imaging, physical examination, and operative findings) and extranodal extension (ENE) or microscopically involved surgical margin lesions (RM+). The intermediate-risk CTV2 encompassed the pathologically positive hemi neck; this frequently required coverage of nodal levels I, IIa-b, III, and IV for most cases. The low-risk CTV3 usually encompassed the prophylactically treated neck with a low risk of harboring microscopic disease (e.g., the uninvolved low or contralateral neck). For the planning target volume (PTV), a 2–5 mm margin was applied to the CTV. The intended total dose for PTV1 was 60–66 Gy in 2.0 Gy per fraction. If ENE or RM+ were present, the region was treated with 64–66 Gy. The intended total doses for PTV2 and PTV3 were 60 Gy and 45-50 Gy, respectively. The target volume was delineated on simulation CT fused with PET and other images. Helical tomotherapy (HT), an image-guided IMRT system using megavoltage CT (MVCT) that provides precise delivery, was used in IMRT. HT was demonstrated to have better target volume dose conformity and homogeneity than other IMRT  (8). The daily MVCT images were fused with the original treatment planning based on soft tissue and bony structures at each fraction. The position was corrected manually to align target volume after automatic registration  (9).

Concurrent chemotherapy was added to RT in patients with high risk OCSCC. High risk was defined by ENE, RM+, perineural invasion (PNI), lymphovascular invasion (LVI), and/or multiple nodes involvement. ENE was defined as extension of cancer cells through the lymph node capsule. The pathological margins were classified as negative (>5 mm), close (≤5 mm), and positive (presence of cancer cells microscopically [RM+]). Patients received concurrent chemotherapy as follows: cisplatin was administered as a weekly dose of 25–40 mg/m^2^ or a triweekly dose of 100 mg/m^2^ from the first day of RT.

Each patient was examined by a dental team for pre-radiotherapy dental care, which was completed before the initiation of PORT. In addition to clinical examination, radiographic examination was performed to determine the periodontal status and the presence of periapical inflammation and other dental diseases. Each patient was examined at least once a week to monitor treatment-related toxicities. Treatment-related toxicities were graded according to the Common Toxicity Criteria for Adverse Events version 5.0.

Patterns of first failure were defined as loco-regional failure or distant metastases. The date of failure was the date of tissue confirmation or imaging study showing evidence of failure. Local failure was defined as failure occurring within the same site of the primary tumor, regional failure if occurring within the regional lymph nodes, and distant failure if occurring outside of the local and regional areas.

### Statistical Analysis

Statistical analyses were conducted using IBM SPSS version 25.0 (IBM Corp., Armonk, NY). The differences in characteristics and toxicities were compared using chi-square tests, and the Kaplan–Meier method was used to calculate the OS, progression-free survival (PFS), loco-regional failure-free survival and distant metastasis-free survival; differences between the curves were analyzed using the log-rank test. Cox proportional hazards models were used to assess the association of variables with the survival and hazard ratio (HR) and confidence interval (CI). Statistical significance was defined as *p* < 0.05. Factors showing *p* < 0.10 in the univariate analyses were included in the multivariate analyses.

## Results

### Patient Characteristics

The patient and pathologic characteristics are summarized in **Table 1**. The median age of patients was 58 years (range, 18–86) and the male-to-female ratio was 6:4. The most common primary site was the oral tongue (54%, n = 148), followed by the buccal mucosa (13%, n = 37), retromolar trigone (12%, n = 32), and alveolar ridge (12%, n = 32). The most common pathologic T and N status were T4 (36%, n = 98) and N3 (30%, n = 82). A total of 119 patients (43%) had stage IVA disease according to American Joint Committee On Cancer (AJCC) 8^th^ edition and 82 patients (30%) had stage IVB disease. A total of 103 patients (37%) had ENE and 72 patients (26%) had RM+.

**Table 1:** Patient characteristics.

Variables	Total (n = 275)	CCRT (n = 148)	RT alone (n = 127)	*p* value
Age (median in year)	58 (18-86)	58.5 (18-80)	58.0 (24–86)	
Age (year)				0.904
<60	143 (52.0)	76 (51.4)	67 (52.8)	
≥60	132 (48.0)	72 (48.6)	60 (47.2)	
Sex				0.900
Male	175 (63.6)	95 (64.2)	80 (63.0)	
Female	100 (36.4)	53 (35.8)	47 (37.0)	
Performance status				0.478
ECOG PS 0–1	256 (93.1)	136 (91.9)	120 (94.5)	
ECOG PS 2	19 (6.9)	12 (8.1)	7 (5.5)	
Subsite				0.960
Tongue	148 (53.8)	79 (53.4)	69 (54.3)	
Buccal mucosa	37 (13.5)	20 (13.5)	17 (13.4)	
Retromolar trigone	32 (11.6)	19 (12.8)	13 (10.2)	
Gingiva, alveolar ridge	32 (11.6)	15 (10.1)	17 (13.4)	
Floor of mouth	18 (6.5)	11 (7.4)	7 (5.5)	
Hard palate	8 (2.9)	4 (2.7)	4 (3.2)	
Pathologic T classification				0.157
T1	56 (20.4)	24 (16.2)	32 (25.2)	
T2	82 (29.8)	43 (29.1)	39 (30.7)	
T3	38 (13.8)	20 (13.5)	18 (14.2)	
T4	99 (36.0)	61 (41.2)	38 (29.9)	
Pathologic N classification				<0.001
N0	70 (25.5)	20 (13.5)	50 (39.4)	
N1	46 (16.7)	19 (12.8)	27 (21.3)	
N2	77 (28.0)	40 (27.0)	37 (29.1)	
N3	82 (29.8)	69 (46.6)	13 (10.2)	
AJCC 8th stage				<0.001
I	12 (4.4)	2 (1.4)	10 (7.9)	
II	22 (8.0)	5 (3.4)	17 (13.4)	
III	40 (14.5)	12 (8.1)	28 (22.0)	
IVA	119 (43.3)	60 (40.5)	59 (46.5)	
IVB	82 (29.8)	69 (46.6)	13 (10.2)	
Extranodal extension				<0.001
Yes	103 (37.5)	83 (56.1)	20 (15.7)	
No	172 (62.5)	65 (43.9)	107 (84.3)	
Lymphovascular invasion				0.033
Yes	59 (21.5)	42 (28.4)	17 (13.4)	
No	216 (78.5)	106 (71.6)	110 (86.6)	
Perineural invasion				0.176
Yes	97 (35.3)	63 (42.6)	34 (26.8)	
No	178 (64.7)	85 (57.5)	93 (73.2)	
Resection margin				0.017
Positive	72 (26.2)	47 (31.8)	25 (19.7)	
Close	94 (34.2)	53 (35.8)	41 (32.3)	
Negative	109 (39.6)	48 (32.4)	61 (48.0)	
RT dose, mean (range, Gy)	61.6 (50.0–67.5)	62.2 (53.0–67.5)	60.9 (50.0–66.0)	0.002
RT modality				0.004
3D-CRT	239 (86.9)	137 (92.6)	102 (80.3)	
IMRT	36 (13.1)	11 (7.4)	25 (19.7)	

ECOG, Eastern Cooperative Oncology Group; AJCC, American Joint Committee on Cancer; RT, radiotherapy; 3D-CRT, 3-dimensional conformal radiotherapy; IMRT, intensity modulated radiotherapy.

The characteristics that differed by treatment group included pathologic N status, AJCC stage, ENE, resection margin status, RT dose and RT modality. More patients in the CCRT group had N3 disease and AJCC stage IVB disease (46.6% *vs.* 10.2%, *p*<0.001), ENE (56.1% *vs.* 15.7%, *p*<0.001), LVI (28.4% *vs.* 13.4%, *p*=0.033). The mean RT dose was higher in the CCRT group (62.2 Gy *vs.* 60.9 Gy, *p*=0.002) and fewer patients received IMRT (7.4% *vs.* 19.7%, *p*=0.004). The other characteristics were well balanced between treatment groups.

Ipsilateral neck dissection was performed for 268 patients (98%), of whom 129 (47%) received modified radical neck dissection (mRND), 121 (44%) received supra-omohyoid neck dissection (SOND), and 18 (7%) received selective neck dissection (SND). Contralateral neck dissection was performed for 87 patients (32%), of whom 10 (4%) received mRND, 58 (21%) received SOND, and 19 (7%) received SND.

A total of 36 patients (13%) received 3D-CRT and 239 patients (87%) received IMRT. The median RT dose was 63 Gy (range, 50–67.5), median high risk CTV fractional dose was 2.1 Gy (range, 1.8–2.5) and median fraction number was 30 (range, 20-36). The median treatment day of RT was 42 days (range, 32–70) and total treatment time from surgery to finish of RT was 82 days (range, 63–177). A total of 148 patients (54%) received concurrent chemotherapy, three patients received induction chemotherapy consisting of cisplatin and gimeracil, also known as TS-1, before surgery, and one patient received maintenance gimeracil chemotherapy after RT. The median cumulative dose of cisplatin was 200 mg/m^2^ (range, 40–300 mg/m^2^). Most patients (66%, n = 97) completed chemotherapy without interruption. A total of 17 patients (12%) discontinued concurrent chemotherapy because of grade 3 poor oral intake (n = 7), grade 3 fatigue (n = 5), and grade 3 neutropenia (n = 5). Furthermore, the chemotherapy dose was reduced in 15 (10%) patients because of grade 3 fatigue (n = 8), and grade 3 neutropenia (n = 7).

### Survival Analysis, Prognostic Factors

**Figure 1:**
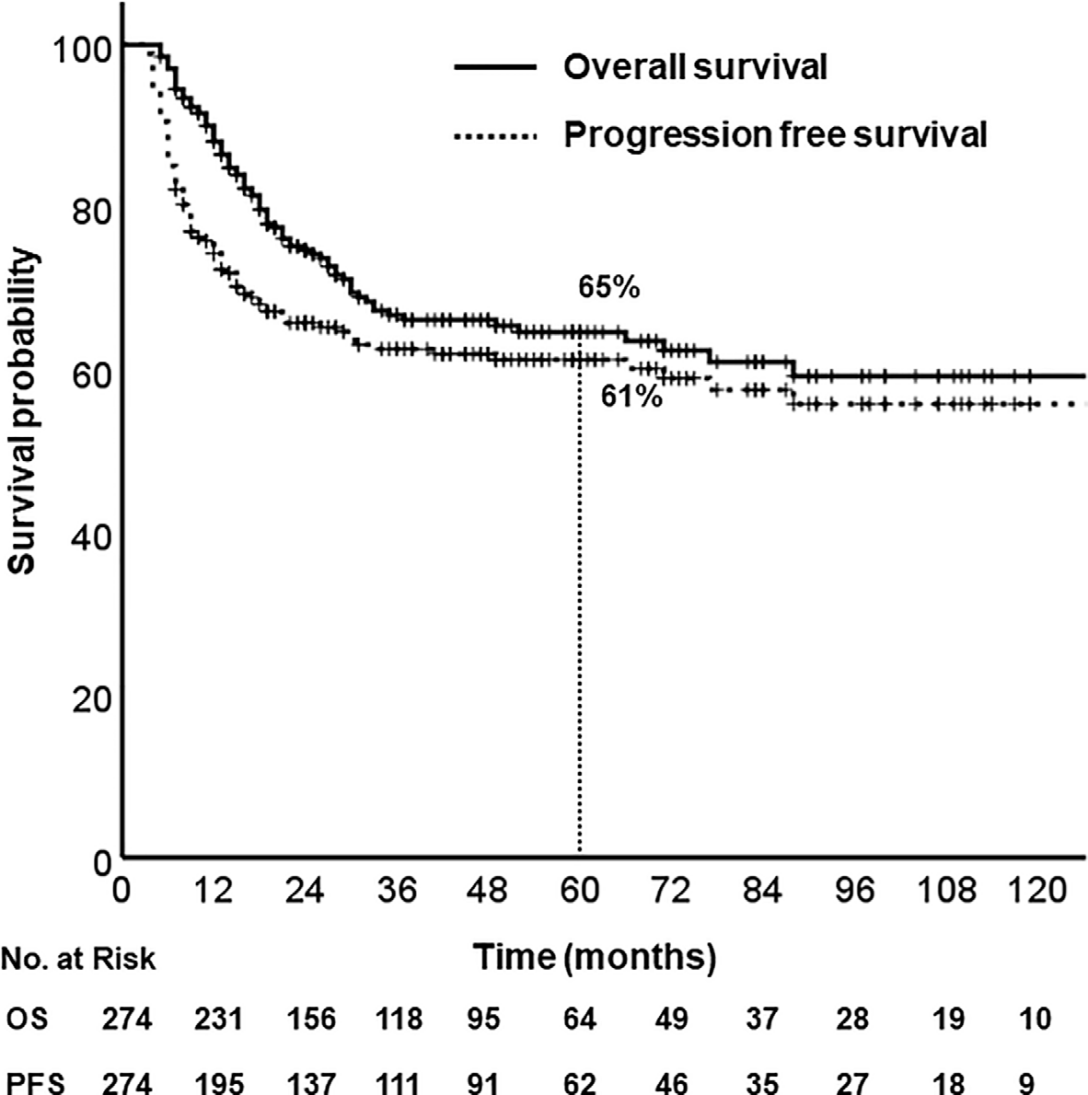
Overall survival and progression free survival.

With a median follow-up of 40 months (range, 5–203), the 5-year OS and PFS rates were 65% and 61%, respectively (**Figure 1**). Median OS was not reached, and median PFS was 140 months (95% CI, 67.6–212.4). According to primary site, the 5-year OS were follows: oral tongue (69%), buccal mucosa (64%), retromolar trigone (55%), gingiva and alveolar ridge (56%), floor of mouth (76%), hard palate (42%).

The prognostic factors associated with OS are summarized in **Table 2**. Univariate analysis revealed that pathologic T and N status, AJCC stage, ENE, and PNI were significant prognostic factors associated with OS. In the multivariate analysis, pathologic T status and PNI were associated with poorer OS. The prognostic factors associated with PFS are summarized in **Table 3**. Univariate analysis revealed that pathologic T and N status, AJCC stage, ENE, and PNI were significant prognostic factors associated with PFS. In the multivariate analysis, pathologic T status and PNI were associated with poorer PFS.

**Table 2:** Prognostic factors for overall survival.

Variable	Univariate			Multivariate		
	HR	95% CI	P value	HR	95% CI	P value
Age (<60 *vs.* ≥60)	1.031	0.673–1.579	0.888			
Sex (Female *vs.* Male)	0.974	0.622–1.525	0.908			
Performance (ECOG PS0–1 *vs.* PS2)	0.857	0.347–2.117	0.739			
T classification (T1–2 *vs.* T3–4)	1.800	1.163–2.786	0.008	1.660	1.043–2.642	0.033
N classification (N0–1 *vs.* N2–3)	1.972	1.230–3.163	0.005	1.348	0.694–2.615	0.378
AJCC stage (I–II *vs.* III–IV)	4.372	1.381–13.840	0.012	2.207	0.620–7.852	0.221
ENE (No *vs.* Yes)	2.065	1.348–3.164	0.001	1.525	0.848–2.740	0.159
LVI (No *vs.* Yes)	1.511	0.942–2.425	0.087	1.234	0.757–2.012	0.399
PNI (No *vs.* Yes)	1.868	1.217–2.867	0.004	1.595	1.020–2.495	0.041
RM (Negative *vs.* Close/Positive)	1.481	0.945–2.322	0.087	1.445	0.916–2.277	0.113
Treatment modality (RT alone *vs.* CCRT)	0.993	0.648–1.522	0.974			
RT modality (3D-CRT *vs.* IMRT)	0.869	0.494–1.530	0.627			

The foreparts of the parentheses were set as the reference group.

HR, hazard ratio; CI, confidence interval; ECOG, Eastern Cooperative Oncology Group; AJCC, American Joint Committee on Cancer; ENE, extranodal extension; LVI, lymphovascular invasion; PNI, perineural invasion; RM, resection margin; RT, radiotherapy; CCRT, concurrent chemoradiotherapy; 3D-CRT, three-dimensional conformal radiation therapy; IMRT, intensity-modulated radiation therapy.

**Table 3:** Prognostic factors for progression free survival.

Variable	Univariate			Multivariate		
	HR	95% CI	P value	HR	95% CI	P value
Age (<60 *vs.* ≥60)	0.930	0.630*–*1.373	0.715			
Sex (Female *vs.* Male)	0.938	0.621*–*1.416	0.761			
Performance (ECOG PS0*–*1 *vs.* PS2)	1.385	0.698*–*2.747	0.352			
T classification (T1*–*2 *vs.* T3*–*4)	1.918	1.287*–*2.857	0.001	1.881	1.224*–*2.891	0.004
N classification (N0*–*1 *vs.* N2*–*3)	2.056	1.338*–*3.159	0.001	1.659	0.925*–*2.975	0.089
AJCC stage (I*–*II *vs.* III*–*IV)	3.319	1.347*–*8.180	0.009	1.387	0.495*–*3.891	0.534
ENE (No *vs.* Yes)	1.873	1.270*–*2.762	0.002	1.314	0.791*–*2.183	0.292
LVI (No *vs.* Yes)	1.375	0.885*–*2.137	0.156			
PNI (No *vs.* Yes)	1.683	1.138*–*2.489	0.009	1.572	1.055*–*2.342	0.026
RM (Negative *vs.* Close/Positive)	1.236	0.827*–*1.849	0.301			
Treatment modality (RT alone *vs.* CCRT)	0.963	0.652*–*1.421	0.848			
RT modality (3D-CRT *vs.* IMRT)	0.934	0.544*–*1.603	0.804			

The foreparts of the parentheses were set as the reference group.

HR, hazard ratio; CI, confidence interval; ECOG, Eastern Cooperative Oncology Group; AJCC, American Joint Committee on Cancer; ENE, extranodal extension; LVI, lymphovascular invasion; PNI, perineural invasion; RM, resection margin; RT, radiotherapy; CCRT, concurrent chemoradiotherapy; 3D-CRT, three-dimensional conformal radiation therapy; IMRT, intensity-modulated radiation therapy.

### Outcomes According to Treatment Group

**Figure 2:**
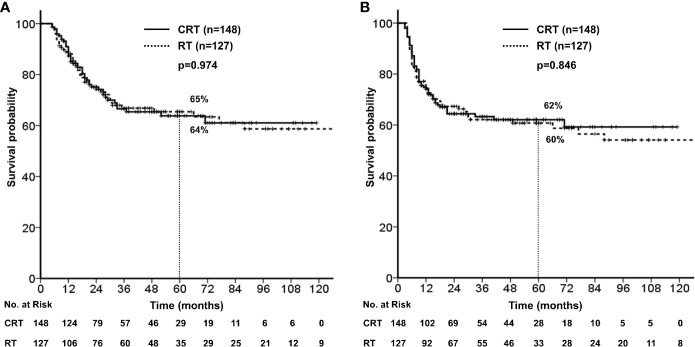
**(A)** Overall survival stratified by concurrent chemotherapy. **(B)** Progression free survival stratified by concurrent chemotherapy.

There were no significant differences in the 5-year OS (64% *vs.* 65%, *p* = 0.974) and PFS (62% *vs.* 60%, *p*=0.846) between treatment groups (**Figure 2**). No significant difference was observed in the 5-year loco-regional failure-free survival (79% *vs.* 77%, *p* = 0.599) and distant metastasis-free survival (78% *vs.* 81%, *p* = 0.475).

**Figure 3:**
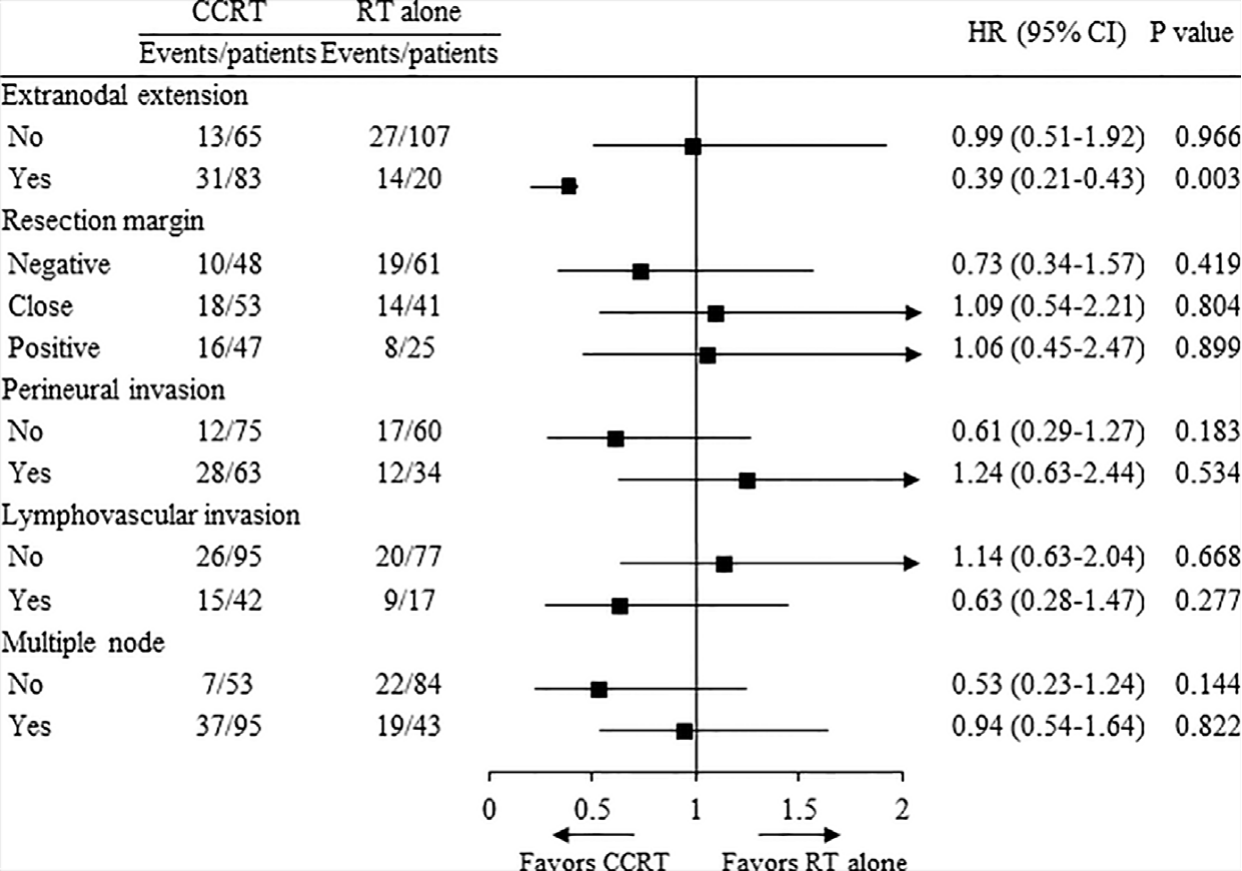
Overall survival in treatment groups.

In the subgroup analysis according to high risk (ENE, RM+, PNI, LVI, and multiple node), the concurrent use of chemotherapy showed significantly improved OS in patients with ENE (HR 0.39, 95% CI 0.21–0.43, *p* = 0.003, **Figure 3**), while there was no advantage in OS in patients with RM+, PNI, LVI, and multiple node.

### Outcomes According to Indications of Chemotherapy

In total, 103 and 72 patients had ENE and RM+, respectively; 22 patients had both ENE and RM+ and 153 patients had either ENE or RM+. Concurrent chemotherapy significantly improved the 5-year OS in patients with ENE (56% *vs.* 32%, *p* = 0.002); however, it did not improve the 5-year OS in patients with RM+ (64% *vs.* 64%, *p* = 0.899). Moreover, concurrent chemotherapy was not beneficial in patients with either ENE or RM+ (*p* = 0.116), but it was beneficial in terms of survival in patients with both ENE and RM+ (*p* < 0.001). The mean RT dose for patients with ENE was statistically higher than that for patients without ENE (62.7 Gy *vs.* 60.9 Gy, *p* < 0.001). Similarly, the mean RT dose for patients with RM+ was statistically higher than that for patients without RM+ (62.6 Gy *vs.* 61.2 Gy, *p* < 0.001).

The most common first pattern of failure in patients with ENE was distant failure (26%), followed by local (16%) and regional failures (15%). In patients with RM+, the rates of local (13%), regional (15%), and distant (13%) failures were similar. The overall rates of distant failure in patients with ENE, RM+, and negative resection margins were 32%, 21%, and 16%, respectively.

### Patterns of First Failure

**Figure 4:**
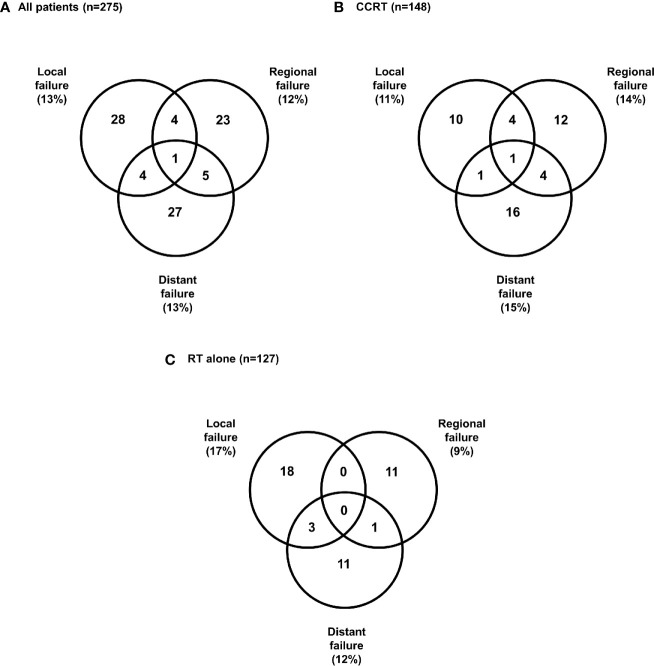
Patterns of first failure **(A)** All patients, **(B)** Concurrent chemoradiotherapy, **(C)** Radiotherapy alone.

Treatment failure occurred in 48 and 59 patients in the RT alone and CCRT groups, respectively. The patterns of first failure are summarized in **Figure 4**. A total of 37 patients (13%) had local failures, 33 patients (12%) had regional failures, and 37 patients (13%) had distant failures (**Figure 4A**). The most common patterns of failure in CCRT was distant failure (15%) followed by regional (14%) and local (11%) (**Figure 4B**), and the most common patterns of failure in RT alone was local failure (17%), followed by distant (12%) and regional failures (9%) (**Figure 4C**).

### Toxicity

A total of 105 patients (38.2%) had grade 3 mucositis, and three patients had grade 3 skin reaction. With respect to late toxicities, 10 patients (3.6%) suffered from osteoradionecrosis (ORN) of the mandible and five patients had orocutaneous fistula. Among the 10 patients with ORN, five patients had a primary tumor near the mandible (one with retromolar trigone and four with gingiva), five had pathologic T4a disease and eight received more than 60 Gy of RT. Among the five patients with orocutaneous fistula, all patients had pathologic T4a disease and received more than 60 Gy, and three had RM+.

## Discussion

This study reports outcomes for patients with OCSCC treated with surgery followed by CCRT or RT alone. A total of 239 patients (87%) were treated with IMRT. With a median follow-up of 40 months, there were no significant differences in the OS and PFS between treatment groups. Considering the fact that patients in the CCRT group had more N3 disease, ENE, and LVI, use of concurrent chemotherapy had beneficial effect on survival than RT alone.

In the EORTC 22931 and RTOG 9501 trials  (6, 7), ENE and/or RM+ were the most significant prognostic factors, and the concurrent chemotherapy with PORT appeared to improve the survival of patients with OCSCC. The 10-year follow-up results were reported to examine long-term outcomes  (6). In the subset analysis limited to patients with ENE and/or RM+, local-regional failure rates were 33.1% *vs.* 21.0% (*p*=0.02) and the OS was 19.6% *vs.* 27.1% (*p*=0.07). These results demonstrated improved disease control with concurrent administration of chemotherapy. In our study, there showed improved survival with concurrent administration of chemotherapy for patients with ENE  (6). In the subgroup analysis, the mean RT dose for patients with ENE or RM+ was statistically higher than that for patients without ENE or RM+. Radiation dose escalation for RM+ could explain the similar rates of local, regional, and distant failures.

Hsieh C.H. et al. reported that PORT with image guidance results in better OS and LCR than postoperative RT without image guidance  (10). Patients who received image-guided IMRT had a better 5-year OS than patients who received non-image-guided IMRT (87% *vs.* 48%). In our institution, all the patients with head and neck cancer received image-guided HT, and patients were verified every day with MVCT. In case of weight loss or change in body shape, we made an adaptive plan for these patients.

According to the report by the Memorial Sloan Kettering Cancer center (MSKCC), 44 of 1,023 patients (4.3%) developed ORN. Patients with ORN had poor periodontal status, a history of heavy alcohol use, and received a higher radiation dose  (11). Patients with oropharyngeal cancer are prone to develop ORN compared to patients with OCSCC because patients with oropharyngeal cancer receive a higher radiation dose. Similar results were observed in our analysis. Among 10 patients with ORN, most of them received a higher radiation dose and had a primary tumor near the mandible. In a report of oropharyngeal cancer from our institution, approximately 30% of patients had overall grade ≥3 acute toxicities treated with definitive RT  (12). In this study, 38% of patients had grade 3 mucositis in the results of OCSCC treated with surgery followed by PORT. With respect to late toxicities, our data showed acceptable results.

Several studies have reported outcomes for specific subsites within the oral cavity. Wang Ling et al. reported the survival outcomes of 210 patients with SCC of the tongue  (13). The 5-year OS rate for patients who underwent surgery and surgery with PORT were 58.2% and 45.6%, respectively. PORT was performed for stage III-IV patients, while stage I-II patients received surgery alone. In our data, the 5-year OS for patients with tongue cancer was 65%. Moreover the MSKCC reported the results of SCC of the gingivobuccal complex  (14). The 5-year OS rate for patients with tongue (n = 936) and gingivobuccal cancer (n = 486) were 67.8% and 61%, respectively. PORT was performed for 40% of tongue cancer patients and 26% of gingivobuccal cancer patients. Patients with gingivobuccal cancer were more likely to be older and have more advanced disease. In our data, the 5-year OS rate was 64% for patients with buccal mucosa cancer. Nishi H. et al. reported 45 patients with retromolar trigone cancer and reported a 3-year OS rate of 59.8%, as well as pathologic LN involvement as a prognostic factor  (15). In our data, the 3-year OS rate for patients with retromolar trigon cancer was 60%. According to a previous retrospective cohort study using the SEER database (Surveillance, Epidemiology, and End Results), the 5-year OS rate for patients with floor of mouth cancer was 40%  (16). In our data, the 5-year OS rate for patients with floor of mouth cancer was 76%, although only 18 patients had floor of mouth cancer.

The current study has several limitations. There was heterogeneity of tumor subsites, surgery techniques including neck dissection methods, and radiation dose and field; these differences may have influenced the local, regional, and distant tumor response. Furthermore, after the first failure, the salvage methods were not uniform. Some patients received re-operation, chemotherapy only, and/or re-irradiation for recurrent tumor; this might have influenced the OS. Because this study was retrospective, the incidence of treatment-related toxicities could be underestimated. However, the current study evaluated a large number of patients with OCSCC who received surgery followed by PORT, and determined the impact of concurrent chemotherapy. Because the EORTC 22931 and RTOG 9501 trials (6, 7) included a heterogeneous group of patients, including those with cancers of the oral cavity, oropharynx, larynx, or hypopharynx, prospective randomized controlled trials are needed for OCSCC. This study could be considered as a preliminary study for such trials. Until such trials have been reported, the recommendation based on the combined analysis of EORTC 22931 and RTOG 9501 still need to be followed  (7).

In conclusion, our retrospective study showed that postoperative CCRT group had comparable survival outcomes to those in the RT alone group for advanced OCSCC in the era of modern RT techniques and indicated that concurrent chemotherapy should be administered to patients with ENE. Prospective randomized studies for confirmation are needed.

## Data Availability Statement

The raw data supporting the conclusions of this article will be made available by the authors, without undue reservation.

## Ethics Statement

The studies involving human participants were reviewed and approved by IRB #4-2019-0401. The patients/participants provided their written informed consent to participate in this study.

## Author Contributions

This study was designed and directed by TK and CL. I-HC, EC, HRK, HJK, S-HK, and KK contributed to the data collection and analysis. The manuscript was written by TK and CL, and commented on by all authors. All authors contributed to the article and approved the submitted version.

## Conflict of Interest

The authors declare that the research was conducted in the absence of any commercial or financial relationships that could be construed as a potential conflict of interest.
